# The effects of physical activity on functional MRI activation associated with cognitive control in children: a randomized controlled intervention

**DOI:** 10.3389/fnhum.2013.00072

**Published:** 2013-03-12

**Authors:** Laura Chaddock-Heyman, Kirk I. Erickson, Michelle W. Voss, Anya M. Knecht, Matthew B. Pontifex, Darla M. Castelli, Charles H. Hillman, Arthur F. Kramer

**Affiliations:** ^1^Department of Psychology, The Beckman Institute for Advanced Science and Technology, University of Illinois at Urbana-ChampaignUrbana, IL, USA; ^2^Department of Psychology, University of PittsburghPittsburgh, PA, USA; ^3^Department of Psychology, The University of IowaIowa City, IA, USA; ^4^Department of Kinesiology, Michigan State UniversityEast Lansing, MI, USA; ^5^Department of Kinesiology and Health Education, The University of Texas at AustinAustin, TX, USA; ^6^Department of Kinesiology and Community Health, University of Illinois at Urbana-ChampaignUrbana, IL, USA

**Keywords:** activation, brain, children, fitness, fMRI, physical activity

## Abstract

This study used functional magnetic resonance imaging (fMRI) to examine the influence of a 9-month physical activity program on task-evoked brain activation during childhood. The results demonstrated that 8- to 9-year-old children who participated in 60+ min of physical activity, 5 days per week, for 9 months, showed decreases in fMRI brain activation in the right anterior prefrontal cortex coupled with within-group improvements in performance on a task of attentional and interference control. Children assigned to a wait-list control group did not show changes in brain function. Furthermore, at post-test, children in the physical activity group showed similar anterior frontal brain patterns and incongruent accuracy rates to a group of college-aged young adults. Children in the wait-list control group still differed from the young adults in terms of anterior prefrontal activation and performance at post-test. There were no significant changes in fMRI activation in the anterior cingulate cortex (ACC) for either group. These results suggest that physical activity during childhood may enhance specific elements of prefrontal cortex function involved in cognitive control.

## Introduction

Physical activity and higher aerobic fitness are associated with improved brain function across the lifespan (Kramer et al., 1999; Hillman et al., 2005, 2009; Davis et al., 2011; Kamijo et al., 2011; Pontifex et al., 2011; Voss et al., 2011; Chaddock et al., 2012a). Prior studies have reported that substantial effects of physical activity occur on tasks that measure cognitive control (Hawkins et al., 1992; Colcombe and Kramer, 2003; Tomporowski et al., 2008), which refers to aspects of cognition that describe the ability to flexibly adapt behavior toward specific goals, maintenance of these goals, monitoring of errors, and formulation of decisions (Botvinick et al., 2001; Braver and Barch, 2006). To achieve high levels of cognitive control, individuals must be able to selectively attend to relevant information, filter distractions, and inhibit inappropriate response tendencies (Bunge and Crone, 2009). Of particular interest to this investigation, higher fit and physically active children have been found to outperform their lower fit peers on tasks of cognitive control [e.g., flanker tasks (Hillman et al., 2009; Pontifex et al., 2011; Voss et al., 2011; Chaddock et al., 2012a), Stroop tasks (Buck et al., 2008), n-back tasks (Kamijo et al., 2011), and real world street crossing multitasking paradigms (Chaddock et al., 2012b)].

Here, functional magnetic resonance imaging (fMRI) was used to examine the influence of a 9-month physical activity program on brain activation patterns associated with cognitive control during childhood. Only a few studies with children have used fMRI to examine how physical activity and aerobic fitness relate to brain function during tasks engaging cognitive control (Chaddock et al., 2011; Davis et al., 2011; Voss et al., 2011). In one study by Davis et al. (2011), overweight children (age 7–13 years) involved in 13 weeks of aerobic games showed improvements in cognitive control (i.e., a “planning” score said to measure strategy and self-regulation) and increases in frontal fMRI activation during an antisaccade task, which provides a measure of response inhibition. However, the interpretation of the fMRI results was constrained because performance on the antisaccade task was not reported. Whereas Davis et al. (2011) suggested that increased frontal activation with physical activity may be associated with improvements in cognitive control, two other studies showed that decreased frontal activation in higher fit children was associated with better cognitive control (Voss et al., 2011; Chaddock et al., 2012a). In a study by Chaddock et al. (2012a), children with higher aerobic fitness levels showed reduced activation in the frontal cortex from early to late stages of a flanker task, coupled with maintenance of attentional and interference control. It is noteworthy that these fitness differences in activation were only apparent for incongruent flanker trials that required substantial cognitive control. During congruent trials, both higher fit and lower fit children showed decreases in activation and maintenance of task performance. In conjunction with these findings, Voss et al. (2011) showed that higher fit children exhibited less activation than lower fit children in a network of brain regions including anterior frontal areas involved in task maintenance and cognitive control, coupled with higher accuracy rates during incongruent flanker task trials that required increased cognitive control. Together, previous studies suggest that physical activity and aerobic fitness influence brain function in regions such as the frontal cortex as well as the ability to adapt neural processes to meet and maintain task goals (Davis et al., 2011; Voss et al., 2011; Chaddock et al., 2012a).

The present study examined brain function, in terms of activation and task performance, during a task of cognitive control in children participating in an after school physical activity intervention compared to children in a wait-list control group. Such a longitudinal design significantly strengthens and extends correlational research on aerobic fitness and childhood brain function (Voss et al., 2011; Chaddock et al., 2012a). Although higher fit and lower fit child groups in previous cross-sectional studies did not differ in variables known to influence cognitive and brain health [e.g., IQ, age, socioeconomic status (SES), pubertal timing, Attention Deficit Hyperactivity Disorder (ADHD)], cross-sectional designs raise the possibility that the observed differences were caused by other unmeasured factors (e.g., genes, personality characteristics, nutrition, intellectual stimulation, etc.). Randomized, controlled trials like the present study are necessary to account for potential selection bias and to establish direct and causal associations among physical activity, aerobic fitness and brain activation patterns in children. Here, children were randomly assigned to either an after school physical activity group or wait-list control group in order to examine how participation in a physical activity program aimed at improving aerobic fitness influences performance on a task of cognitive control *and* brain function associated with cognitive control.

In addition, in the present study, to further strengthen and extend previous research, the brain function of the physical activity intervention children and wait-list control children was compared to the activation of college-aged young adults. Adult task performance and activation patterns are often characterized as the “mature” or “optimal” model of brain function to which children can be compared (Luna et al., 2010). Although fMRI studies of age-related differences in cognitive control report a variety of results (see Luna et al., 2010 for a review), the majority of studies demonstrate increased frontal activity in children relative to adults (e.g., Casey et al., 1997; Durston et al., 2002; Booth et al., 2003; Scherf et al., 2006; Velanova et al., 2008), coupled with poorer task performance during cognitive tasks of inhibition (e.g., Go/NoGo, flanker, antisaccade tasks; Diamond, 2006) and working memory (e.g., n-back, visual spatial working memory; Baddeley, 1986; Bunge and Crone, 2009). Nevertheless, throughout childhood, there are continued improvements in cognitive control, and the frontal cortex plays a primary role in performance changes (Luna et al., 2010). Thus, this study explored how participation in physical activity during childhood influences brain function in frontal brain regions, as well as how the changes mirror patterns of adult activation and cognitive abilities.

The first goal of this study was to determine the brain areas, especially frontal brain regions, in children that were associated with an fMRI task of cognitive control. For example, cognitive control has been associated with frontal regions including (1) the anterior prefrontal cortex, hypothesized to maintain task goals, (2) the lateral prefrontal cortex, hypothesized to initiate flexible adjustments in cognitive control, and play a role in working memory, and (3) the anterior cingulate cortex (ACC), hypothesized to evaluate and monitor conflict and thereby signal the need to adjust control (Hazeltine et al., 2000; Botvinick et al., 2001; Bunge et al., 2002; Braver and Barch, 2006; Dosenbach et al., 2007). The second goal was to examine whether brain function in the regions associated with the task changed from pre-test to post-test in the physical activity intervention group compared to any changes occurring in the wait-list control group. It was hypothesized that children involved in a 9-month physical activity program would show improvements in performance on the task, coupled with decreased activation in frontal brain regions from pre-test to post-test, relative to a wait-list control group. The third goal was to compare the activation patterns of both groups of children to the activation of young adults. It was predicted that the frontal activation patterns and performance of physically active children at post-test would show greater similarity to the brain function of young adults, relative to the post-test patterns of the wait-list control children.

## Methods

### Participants

Eight- to nine-year-old children were recruited from the Urbana, Illinois School District 116. All children completed demographic assessments, a VO_2_ max test to assess aerobic fitness, and an MRI session (which included a structural and functional MRI scan) at pre-test (i.e., before randomization into a physical activity intervention group or a wait-list control group) and post-test (i.e., after the completion of the intervention, approximately 9 months later).

Thirty-two children were eligible for the study. Seven children (three physical activity intervention children four wait-list control children) were excluded from the analyses for excessive motion during the fMRI task. Two children were excluded from the analyses (two physical activity intervention children) for less than chance task performance. Accordingly, 23 children, with pre-test and post-test fMRI data, were included in the final analyses, with 14 children (seven female, seven male) assigned to the physical activity intervention group and nine children (six female, three male) assigned to the wait-list control group. Twenty-four young adults (10 female, 14 male) (mean age of 22.5 years) were also recruited from the University of Illinois to compare children's brain and performance patterns to a young adult group.

### Demographic assessments and fitness testing

To be eligible for the study, children had to have a Kaufman Brief Intelligence Test (KBIT) composite score greater than 85 (Kaufman and Kaufman, 1990) and qualify as prepubescent (Tanner puberty score ≤ 2; Taylor et al., 2001). Children were also screened for the presence of attentional disorders using the ADHD Rating Scale IV (DuPaul et al., 1998), and were excluded if they scored above the 85th percentile. Body mass index (BMI) was calculated as weight (kg)/height(cm)^2^, and SES was determined by creating a trichotomous index: participation in a free or reduced-price meal program at school, the highest level of education obtained by the child's mother and father, and the number of parents who worked full-time (Birnbaum et al., 2002; Hillman et al., 2012).

Eligible children were further required to (1) report an absence of school-related learning disabilities (i.e., individual education plan related to learning), adverse health conditions, physical incapacities, or neurological disorders, (2) report no use of medications that influence central nervous system function, (3) demonstrate right handedness (as measured by the Edinburgh Handedness Questionnaire; Oldfield, 1971), (4) complete a mock MRI session successfully to screen for claustrophobia in an MRI machine, (5) be capable of performing physical activity, and (6) sign an informed assent approved by the University of Illinois at Urbana-Champaign. A legal guardian also provided written informed consent in accordance with the Institutional Review Board of the University of Illinois at Urbana-Champaign. Children were paid for their time ($10/h for demographic assessments and fitness testing and $15/h for MRI testing).

### Aerobic fitness testing

Children completed a VO_2_ max test at pre-test and post-test to assess aerobic fitness. The aerobic fitness of each child was measured as maximal oxygen consumption (VO_2_ max) during a graded exercise test (GXT). The GXT employed a modified Balke Protocol and was administered on a LifeFitness 92T motor-driven treadmill (LifeFitness, Schiller Park, IL). Children walked and/or ran on a treadmill at a constant speed with increasing grade increments of 2.5% every 2 min until volitional exhaustion occurred.

Oxygen consumption was measured using a computerized indirect calorimetry system (ParvoMedics True Max 2400) with averages for VO_2_ and respiratory exchange ratio (RER) assessed every 20 s. A polar heart rate (HR) monitor (Polar WearLink+ 31; Polar Electro, Finland) was used to measure HR throughout the test, and ratings of perceived exertion (RPE) were assessed every 2 min using the children's OMNI scale (Utter et al., 2002). Maximal oxygen consumption was expressed in ml/Kg/min and VO_2_ max was based upon maximal effort as evidenced by (1) a plateau in oxygen consumption corresponding to an increase of less than 2 ml/Kg/min despite an increase in workload; (2) a peak HR ≥ 185 beats per minute (American College of Sports Medicine, 2006) and an HR plateau (Freedson and Goodman, 1993); (3) RER ≥ 1.0 (Bar-Or, 1983); and/or (4) a score on the children's OMNI RPE scale ≥ 8 (Utter et al., 2002).

### Physical activity training intervention and wait-list control group

The physical activity intervention occurred for 2 h after each school day, from September until May, for 150 days out of the 170-day school year. The program, Fitness Improves Thinking in Kids (FIT Kids) (http://clinicaltrials.gov/ct2/show/NCT01334359?term=FITKids&rank=1) is based on the Child and Adolescent Trial for Cardiovascular Health (CATCH) curriculum (McKenzie et al., 1994). CATCH is one of the only evidenced-based physical activity programs to incorporate educational, behavioral, and environmental components (Luepker et al., 1996; Nader et al., 1999), resulting in moderate to vigorous physical activity engagement. Although the primary aim of the program targeted improving aerobic fitness through engagement in a variety of age-appropriate physical activities, it was also designed to meet a child's daily need for physical activity by providing 3 or more days per week of aerobic activity as well as muscle and bone strengthening activities (United States Department of Health and Human Services, 2008). The environment was non-competitive and integrated activities such as fitness activities and low organized games (Castelli et al., 2011).

Within a daily lesson, the children participated in an average of 76.8 min of moderate to vigorous physical activity (recorded by E600 Polar HR monitors; Polar Electro, Finland, and Accusplit Eagle 170 pedometers, San Jose CA), thus exceeding the national physical activity guideline of 60 min of moderate to vigorous physical activity per day (United States Department of Health and Human Services, 2008). Children completed stations that focused on a specific health-related fitness component (e.g., cardiorespiratory endurance, muscular strength, motor skills) and participated in game play. The activities were aerobically demanding, but simultaneously provided opportunities to refine motor skills. The program also included consumption of a healthy snack and the introduction of a themed educational component related to health promotion (i.e., goal setting, self-management). On the weekends, the children were encouraged to continue their participation in physical activity with their family, and physical activity worksheets were utilized during school holidays to log continued engagement. Average attendance across the 9-month intervention was 82% (*SD* = 13.3%).

The wait-list control group was not contacted following randomization. They completed all facets of the pre-test and post-test, similar to those children who were randomized into the after school physical activity intervention. As incentive to stay in the study, children in the wait-list control group were afforded the opportunity to participate in the intervention during the following school year.

### Imaging method

Children and young adults completed structural and functional MRI scans. Prior to scanning, all participants were tested for visual acuity, and corrective lenses were added to MRI safe plastic frames to ensure a corrected vision of at least 20/40 while in the scanner. The lenses and frames did not obstruct a mirror above participants' eyes that enabled them to view images on a back projection.

#### Structural MRI protocol

High resolution T1-weighted brain images were acquired using a 3D MPRAGE (Magnetization Prepared Rapid Gradient Echo Imaging) protocol with 192 contiguous axial slices, collected in ascending fashion parallel to the anterior and posterior commissures, echo time (TE) = 2.32 ms, repetition time (TR) = 1900 ms, field of view (FOV) = 230 mm, acquisition matrix 256 × 256 mm, slice thickness = 0.90 mm, and flip angle = 9°. All images were collected on a Siemens Magnetom Trio 3T whole-body MRI scanner.

#### Functional MRI protocol

**Figure 1:**
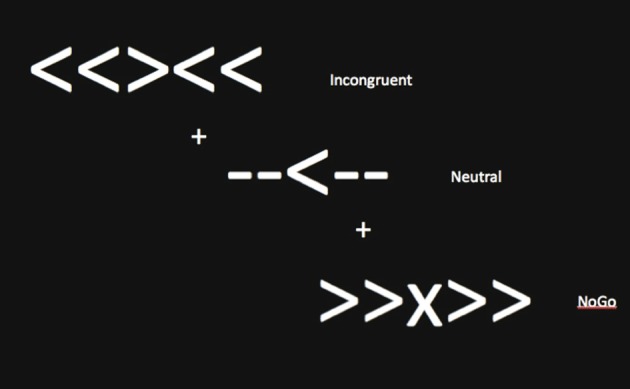
**Sample stimuli for the fMRI task of cognitive control**.

Functional MRI scans were acquired during an event-related cognitive control task. Five shapes were presented, and participants were instructed to look at the middle shape. Three task conditions were included: neutral (-->--, --<--), incongruent (<<><<, >><>>), and NoGo (<<X<<, >>X>>) trials (see Figure 1). When the middle arrow pointed to the left, participants were instructed to press a button with their left index finger. When the middle arrow pointed to the right, participants were instructed to press a button with their right index finger. When the middle shape was an X, participants were told not to press a button. Participants were asked to respond as quickly and accurately as possible. The neutral condition was designed to require less attentional, interference and inhibitory control. The incongruent condition required attentional and interference control to filter potentially misleading flankers that were mapped to incorrect behavioral responses. The NoGo condition required subjects to inhibit a prepotent tendency to respond, given that the majority of trials (i.e., incongruent, neutral) required an active “go” response.

During the task, 40 trials of each of the three possible conditions (-->--, --<--, >><>>, <<><<, >>X>>, <<X<<) were presented in a random order. The response window included the presentation of the array of shapes for 500 ms, followed by a blank screen for 1000 ms. Each stimulus array was separated by a fixation cross (+) presented for 1500 ms. Forty additional fixation crosses that jittered between 1500 and 6000 ms were also randomly presented after the constant 1500 ms fixation cross throughout the task. The jitter prevented participants from expecting a specific frequency of responding. White shapes and white fixation crosses were presented on a black background. The participant was engaged in the task for about 6 min. Stimulus presentation, timing, and task performance measures were controlled by E-Prime software (Psychology Software Tools, Sharpsburg, Pennsylvania).

For the fMRI protocol during the flanker task, a fast echo-planar imaging (EPI) sequence with Blood Oxygenation Level Dependent (BOLD) contrast was employed. A total of 328 volumes (TR = 1500 ms; TE = 25 ms; flip angle = 80°) were collected for each participant.

#### Image analysis

Neuroimaging data analysis was conducted using FSL 4.1.9 (FMRIB's Software Library, www.fmrib.ox.ac.uk/fsl). All child data and young adult data followed the same pre-processing, registration, and first level analysis stream. Preprocessing of the functional data included motion correction via a rigid body algorithm in MCFLIRT (Jenkinson et al., 2002), removal of non-brain structures using BET (Brain Extraction Technique; Smith et al., 2002), spatial smoothing using a 5.0 mm FWHM (full width at half maximum) three-dimensional Gaussian kernel, and temporal filtering with a high pass frequency cut-off of 40 s. In addition, the high-resolution T1 structural images of each participant were skull stripped using BET (Smith et al., 2002). The functional images of each participant were spatially registered to his/her individual skull-stripped high-resolution anatomical image, and then to an MNI template in stereotaxic space. Registrations were conducted using a 12-parameter affine transformation [FMRIB's Linear Image Registration Tool (FLIRT); (Jenkinson and Smith, 2001; Jenkinson et al., 2002)].

Regression-based analysis of each participant's fMRI data was carried out using FSL's FEAT Version 5.98 (Beckmann et al., 2003). The time series at each voxel was modeled against the expected time series model derived by convolving the onset of each event type (incongruent, neutral, NoGo) with a double-gamma function, representing the expected time course of the hemodynamic response function. Only correct task trials were included in the model, and error trials were entered as covariates of no interest. The same high pass temporal filtering applied to the data was applied to the general linear model for the best possible match between the data and model. In addition, the temporal derivative was entered into the model (i.e., shifting the waveform slightly in time) to achieve a better model fit to the data and to reduce unexplained noise. The first level analysis calculated a parameter estimate for the fMRI model at each voxel to estimate how strongly the model waveform fits the data, and this analysis resulted in voxel-wise statistical parametric maps for the entire brain of each participant for each task condition.

Next, the brain maps of all children at pre-test and post-test were forwarded to a higher-level mixed-effects group analysis to localize areas of cortex in all child participants at pre-test and post-test that were activated during the task of cognitive control. Higher-level group analyses were carried out using FLAME (FMRIB's Local Analysis of Mixed Effects). To ensure that individual and group differences in gray matter volume did not confound the results, estimated total mean gray matter volume for each child at pre-test and post-test, smoothed with 3 mm HWHM kernel, was used as a voxel-wise covariate in the higher level FLAME analyses.

A Z statistic map that showed average activation during all task conditions relative to fixation baseline was created for all children, across pre-test and post-test. This conjunction map was used to locate and extract ROIs so that the regions would be chosen independently of effects associated with group (physical activity intervention, wait-list control), task condition (incongruent, neutral, NoGo), and time (pre-test, post-test). This technique helped ensure that the localization of the ROIs was unbiased in relation to the predictor variables.

**Figure 2:**
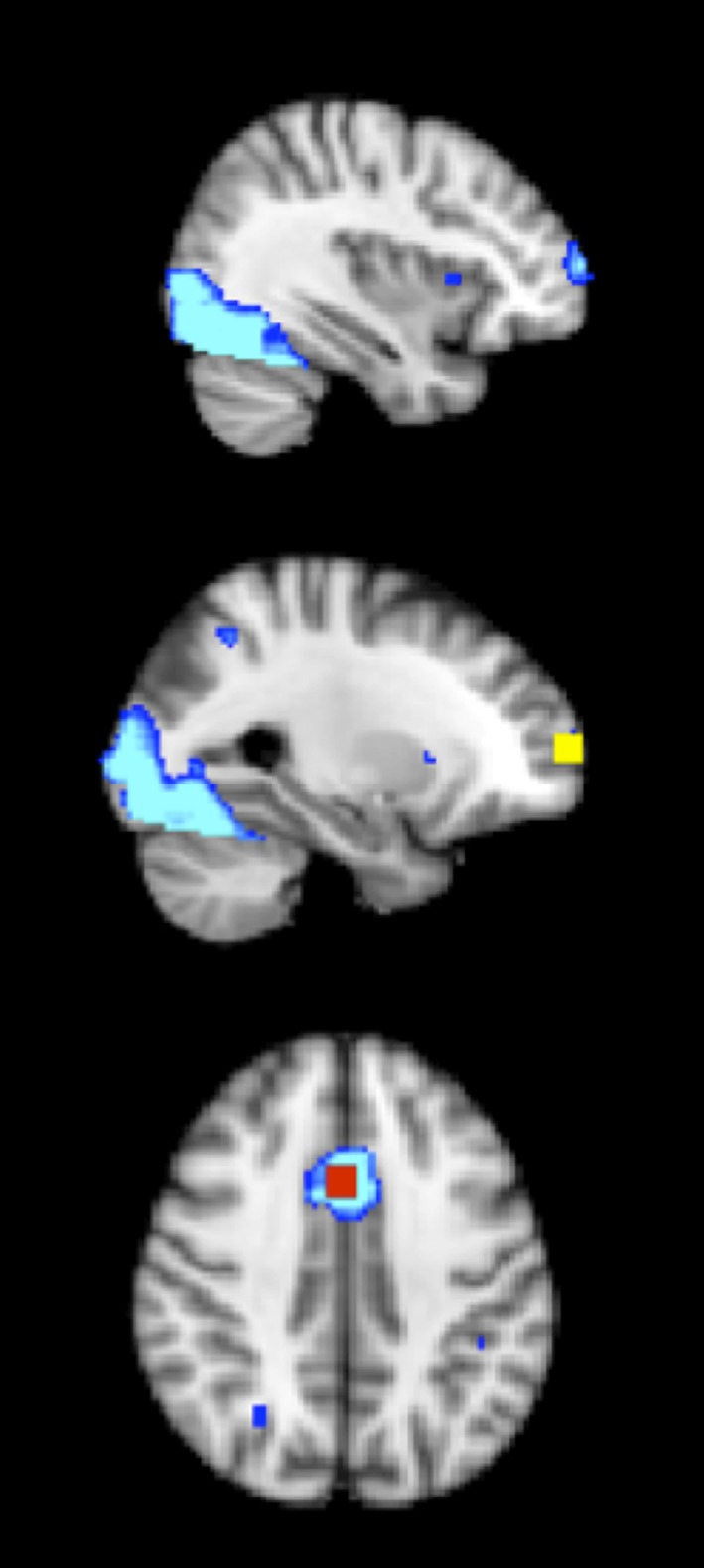
**The 8 mm (diameter) box ROIs (1000 mm^3^) in the frontal cortex, derived from an average activation map during incongruent, neutral and NoGo conditions of the task of cognitive control, across both physical activity and control child groups at pre-test and post-test (thresholded at *Z* > 6).** Right anterior prefrontal cortex = yellow; ACC = red; average activation map = blue.

Because of the widespread activation for the task, a more conservative statistical threshold to identify clusters for the regions-of-interest analysis was employed (Kriegeskorte et al., 2009). Accordingly, the *Z* statistic maps were thresholded at *Z* > 6.00, with a (corrected) cluster significance threshold of *p* < 0.05 (Worsley, 2001). Of particular interest, two clusters in the frontal cortex were observed: (1) the right anterior prefrontal cortex (right frontal pole) (with *x*, *y*, *z* voxel coordinates of 27, 94, 40, *Z* = 6.2) (see Figure 2), and (2) the ACC (with *x*, *y*, *z* voxel coordinates of 44, 69, 55, *Z* = 7.1) (see Figure 2). Eight millimeter (diameter) masks (which contained 125 voxels, 1000 mm^3^) were created around each peak to use as functionally defined ROIs (see Figure 2). Mean percent signal change (versus fixation) was extracted for incongruent, neutral, and NoGo conditions. Note that some activation was also seen in the insula and occipital lobe, but these areas are not a focus of the paper given lack of effects and lack of hypotheses in these areas.

### Statistical analysis

Multivariate repeated measures ANOVAs were first conducted to explore changes in aerobic fitness (VO_2_ max) and task performance in the physical activity and wait-list control groups from pre-test to post-test. Given *a priori* hypotheses, paired *t*-tests were also conducted to compare within-group changes in fitness and task performance. In addition, task performance of the physical activity intervention group and wait-list control group at pre-test and post-test were compared to the young adult group.

Next, the peaks of activation in the brain during the fMRI task of cognitive control, in all children, across task conditions, at pre-test and post-test were determined. Within these regions of interest (ROIs), repeated measures 2 (group: physical activity intervention, wait-list control) × 3 (task condition: incongruent, neutral, NoGo) × 2 (time: pre-test, post-test) ANOVAs were conducted to explore changes in mean percent signal change in the ROIs. If the omnibus ANOVA reached significance, *post-hoc* comparisons were performed (with Bonferroni-corrected *t*-tests) to examine how activation patterns within each task condition changed with participation in physical activity or assignment to a wait-list control group. Further, independent *t*-tests were conducted between the physical activity intervention group, wait-list control group, and young adults at pre-test and post-test to explore how changes in activation over time in children compared to activation in a young adult sample. The family-wise alpha level was set at *p* = 0.05.

## Results

### Participant demographics and aerobic fitness

Demographic and fitness data at pre-test and post-test are provided in Table 1. Demographic and fitness variables of age, gender, race, KBIT (IQ), SES, pubertal timing, and VO_2_ max, did not differ between the physical activity intervention group and the wait-list control group (all *p* > 0.05).

**Table 1:** **Mean (SD) for physical activity and control groups at pre-test and post-test**.

	**Physical activity**		**Control**	
	**Pre-test**	**Post-test**	**Pre-test**	**Post-test**
Age (years)	8.9 (0.7)	9.6 (0.7)	8.9 (0.4)	9.5 (0.5)
Gender	7 girls, 7 boys		6 girls, 3 boys	
IQ	122.3 (14.9)	122.6 (11.8)	114.4 (16.2)	116.4 (18.7)
Pubertal timing	1.3 (0.4)	1.5 (0.4)	1.1 (0.2)	1.5 (0.8)
SES	2.2 (0.9)	2.3 (0.9)	1.8 (0.9)	1.7 (0.9)
VO_2_ max (ml/Kg/min)	38.3 (4.0)	40.6 (4.1)	37.3 (6.2)	39.0 (4.5)
VO_2_ max percentile	14.0 (14.9)^a^	20.0 (18.6)^a^	14.8 (12.5)	16.9 (13.5)
BMI (kg/cm^2^)	18.4 (3.6)	18.7 (4.4)	19.3 (3.7)	20.0 (3.4)

Note: IQ, composite standardized score of intelligence quotient from the Kaufman Brief Intelligence Test (Kaufman and Kaufman, 1990), SES, socioeconomic status. Values that share a common superscript are significantly different at p < 0.05.

The physical activity intervention group showed a 6% increase in VO_2_ max percentile from pre-test to post-test [*t*_(13)_ = 2.0, *p* = 0.06], and the wait-list control group showed a 2% increase in VO_2_ max percentile [*t*_(8)_ = 1.0, *p* = 0.3]. There was also a marginal effect of time for VO_2_ max percentile [*F*_(1, 21)_ = 4.1, *p* = 0.057], but no group × time interaction [*F*_(1, 29)_ = 0.9, *p* = 0.3]. Together, the data suggest an increase in VO_2_ max in all children with age and development (Janz and Mahoney, 1997). However, the physical activity intervention group showed additional within-group gains in VO_2_ max as a function of their daily exposure to physical activity.

### Task performance

All task performance data for the physical activity intervention group, the wait-list control group, and the young adults are in Table 2. To confirm the efficacy of the task, performance differences in all children during the three task conditions were explored. In general, the performance data suggested that the incongruent flanker task provided the greatest challenge to the participants' ability to pay attention, suppress distraction, and maintain a task set. That is, shorter reaction time (RT) for neutral trials (*M* = 805.5 ms, *SD* = 130.4 ms) compared to incongruent trials (*M* = 875.0 ms, *SD* = 152.0 ms) was found at both pre-test and post-test [main effect of task condition, *F*_(1, 21)_ = 44.1, *p* < 0.001]. Higher accuracy for neutral trials (*M* = 94.7%, *SD* = 0.05) compared to incongruent trials (*M* = 90.3%, *SD* = 0.07%) was also found at both pre-test and post-test [*t*_(22)_ = 6.1, *p* < 0.001] [main effect of task condition, *F*_(2, 21)_ = 42.2, *p* < 0.001].

**Table 2:** **Mean task performance (SD) (range) for the physical activity (PA) and control (C) groups at pre-test and post-test, as well as the young adult group**.

	**PA pre-test**	**PA post-test**	**C pre-test**	**C post-test**	**Young**
Incongruent RT (ms)	919.4 (177.3) (658.3–1246.7)^a^^**^	801.5 (173.7) (602.2–1163.0)^a^^**^	937.3 (108.6) (793.4–1166.7)	841.8 (132.6) (663.6–1141.3)	606.1 (86.9) (464.6–760.1)
Neutral RT (ms)	826.6 (139.6) (652.3–1072.6)^a^^**^	755.6 (157.7) (585.8–1108.3)^a^^**^	850.1 (84.1) (693.7–975.4)	789.6 (132.3) (649.6–1098.5)	551.8 (83.1) (413.5–708.7)
Incongruent accuracy (% correct)	85.9 (9.8) (70.0–98.0)^a^^*^	91.2 (4.9) (85–100)^a^^*^	83.9 (14.8) (60.0–100.0)	86.9 (9.9) (70.0–98.0)	92.6 (4.6) (78.0–100)
Neutral accuracy (% correct)	91.6 (6.2) (78.0–100)^a^^**^	95.7 (3.5) (88.0–100)^a^^**^	89.2 (12.6) (63.0–100)	91.1 (5.7) (83.0–100)	96.5 (1.9) (93.0–100)
NoGo accuracy (% correct)	98.8 (1.6) (95.0–100)	98.9 (2.1) (93.0–100)	98.6 (1.8) (95.0–100)	98.6 (1.8) (95.0–100)	98.9 (2.2) (93.0–100)

Note: Values that share a common superscript are significantly different at

** p < 0.05.

* p < 0.1. Effect sizes (Cohen's d): PA pre-test to post-test changes: incongruent RT-ES = 0.67, neutral RT-ES = 0.48, incongruent accuracy-ES = 0.68, neutral accuracy-ES = 0.81, NoGo accuracy-ES = 0.05. C pre-test to post-test changes: incongruent RT-ES = 0.78, neutral RT-ES = 0.54, incongruent accuracy-ES = 0.23, neutral accuracy-ES = 0.19, NoGo accuracy-ES = 0.

However, inconsistent with predictions, children performed at near ceiling accuracy rates during the NoGo task condition. NoGo accuracy (*M* = 98%, *SD* = 1.5%) was significantly higher than incongruent accuracy (*M* = 87%, *SE* = 1.6%) [*t*_(22)_ = 7.3, *p* < 0.001] and neutral accuracy (*M* = 92%, *SD* = 7.7%) [*t*_(22)_ = 5.0, *p* < 0.001] [main effect of task condition, *F*_(2, 21)_ = 42.2, *p* < 0.001]. These results suggest that the NoGo task condition was not sufficiently difficult to yield group differences in response inhibition, or that 8- and 9-year-old children may have more “mature” response inhibition abilities than interference control skills (Bunge et al., 2002; van den Wildenberg and van der Molen, 2004; Liston et al., 2006; Bunge and Crone, 2009). The task design may also have affected performance outcomes. In most Go/NoGo paradigms, participants press a button on the go trials and must override this prepotent response when a NoGo trial appears. However, in this modified task presented herein, participants had to analyze each stimulus array to determine whether they should press a left button, a right button, or withhold their response. Thus, children in this study were unlikely to have developed a prepotent response tendency because they were unable to plan a motor response until the stimulus appeared. This may have led to the high performance across all children on the NoGo trials. Given these limitations, the present study focuses on results regarding incongruent and neutral flanker task conditions that required different amounts of cognitive control.

Improvements in task performance after 9 months were found for all children, which were predicted with development and practice. Shorter post-test RT across incongruent and neutral trials (*M* = 797.1 ms, *SD* = 155.9 ms) was found relative to pre-test RT (*M* = 883.4 ms, *SD* = 135.2 ms) [main effect of time, *F*_(1, 21)_ = 18.8, *p* < 0.001], with larger changes from pre-test to post-test for incongruent RT [*M* = 109.1 ms, *SD* = 101.6 ms; effect size (ES) = 3.18] compared to neutral RT (*M* = 66.7 ms, *SD* = 92.5 ms; *ES* = 2.27) [condition × time interaction, *F*_(1, 21)_ = 8.2, *p* = 0.009]. The main effect of time was only marginally significant for accuracy [*F*_(1, 42)_ = 2.9, *p* = 0.1], which suggested only modest increases in task accuracy for all children from pre-test (*M* = 91.3%, *SE* = 7.2%) to post-test (*M* = 93.8%, *SE* = 0.8%).

#### Physical activity and wait-list control groups

The group × condition × time interaction did not reach significance for RT [*F*_(1, 21)_ = 0.17, *p* = 0.68] or accuracy [*F*_(1, 42)_ = 0.29, *p* = 0.59]. Change scores (difference between post-test performance and pre-test performance) were also calculated for the physical activity intervention group (PA) and wait-list control group (C) (Incongruent RT—PA: *M* = 117.8 ms, *SD* = 71.4 ms, C: *M* = 95.5 ms, *SD* = 140.5 ms; Neutral RT—PA: *M* = 71.0 ms, *SD* = 80 ms, C: 60.4 ms, *SE* = 113.3 ms; Incongruent accuracy—PA: *M* = 5.3%, *SD* = 10.6%, C: *M* = 3.1%, *SD* = 16.7%; Neutral accuracy—PA: *M* = 4.3%, *SD* = 5.8%, C: *M* = 2.0%, *SD* = 9.4%; NoGo accuracy—PA: *M* = 0.1%; *SD* = 2.3%, C: *M* = 0.0%, *SD* = 1.8%), but independent *t*-tests did not yield significant differences between the groups (all *t* < 0.7, *p* > 0.4).

**Figure 3:**
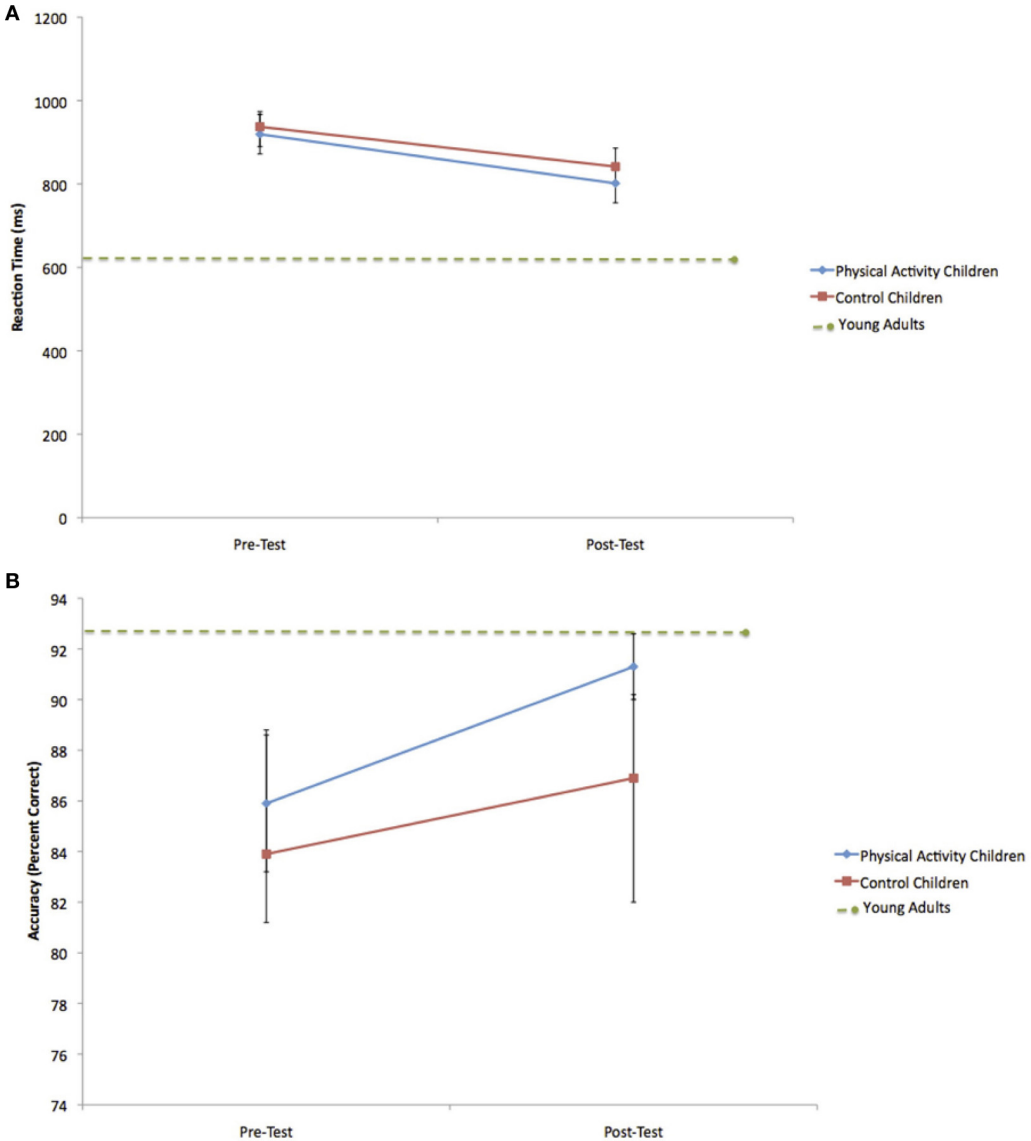
**(A)** Change in incongruent RT for the physical activity intervention group and wait-list control group. Error bars represent standard error. The child groups showed longer RT at pre-test and post-test compared to young adults. **(B)** Change in incongruent accuracy for the physical activity intervention group and wait-list control group. The within-group increase in accuracy for the physical activity intervention group led to accuracy rates at post-test that did not differ from young adults. Error bars represent standard error.

Because of *a priori* hypotheses about greater changes in task performance for the physical activity intervention group relative to the wait-list control group, paired *t*-tests were conducted to further explore the data. Consistent with hypotheses, the physical activity intervention group showed shorter RT for both incongruent trials [*t*_(13)_ = 6.2, *p* < 0.001] and neutral trials [*t*_(13)_ = 3.3, *p* = 0.006] at post-test relative to pre-test (see Table 2 and Figure 3A). The physical activity intervention group also showed a trend for increased accuracy for incongruent trials [*t*_(13)_ = 1.9, *p* = 0.08] (see Figure 3B) and a significant increase in accuracy for neutral trials [*t*_(13)_ = 2.5, *p* = 0.03] at post-test relative to pre-test, but no changes in accuracy for NoGo trials [*t*_(13)_ = 0.3, 0.8] (see Table 2). Alternatively, the wait-list control group showed a trend for shorter RT from pre-test to post-test [incongruent: *t*_(8)_ = 2.1, *p* = 0.08; neutral: *t*_(8)_ = 1.6, *p* = 0.15], but no significant changes in accuracy from pre-test to post-test (all *t* < 0.7, all *p* > 0.6) (see Table 2). The data raise the possibility that the physical activity intervention group was responsible for some of the general performance improvements across all children from pre-test to post-test.

#### Children and young adults

To gain more insight into changes in task performance within the physical activity intervention group and wait-list control group, the pre-test and post-test task performance for the groups of children were compared to a group of young adults (see Table 2). It was predicted that children and young adults would differ in task performance at pre-test, due to age effects, but participation in physical activity may reduce the age effects at post-test.

As predicted, at pre-test, the physical activity intervention group and wait-list control group showed longer RT than young adults during incongruent [PA: *t*_(36)_ = 7.3, *p* < 0.001; C: *t*_(31)_ = 9.1, *p* < 0.001] and neutral [PA: *t*_(36)_ = 7.7, *p* < 0.001; C: *t*_(31)_ = 5.6, *p* < 0.001] trials. Both groups of children also showed lower accuracy rates during incongruent [PA: *t*_(36)_ = 2.8, *p* = 0.03; C: *t*_(31)_ = 2.6, *p* = 0.01] and neutral [PA: *t*_(36)_ = 3.6, *p* = 0.01; C: *t*_(31)_ = 2.8, *p* = 0.008] trials.

However, at post-test, the physical activity intervention group did not differ from young adults in terms of incongruent accuracy [*t*_(36)_ = 0.7, *p* = 0.5] (see Figure 3B) or neutral accuracy [*t*_(36)_ = 0.9, *p* = 0.4]. Alternatively, the wait-list control group still showed lower accuracy rates than young adults during incongruent [*t*_(31)_ = 2.2, *p* = 0.04] and neutral [*t*_(31)_ = 4.1, *p* < 0.001] task trials. In terms of RT, both the physical activity intervention and wait-list control groups showed longer RT than young adults during incongruent [PA: *t*_(36)_ = 4.7, *p* < 0.001; C: *t*_(31)_ = 9.2, *p* < 0.001] (see Figure 3A) and neutral [PA: *t*_(36)_ = 5.2, *p* < 0.001; C: *t*_(31)_ = 6.2, *p* < 0.001] trials at post-test. No age-related performance differences between the physical activity intervention group, wait-list control group and young adults were found for NoGo trials at pre-test [PA: *t*_(36)_ = 0.2, *p* = 0.9; C: *t*_(31)_ = 0.3, *p* = 0.7] or post-test [PA: *t*_(36)_ = 0.1, *p* = 0.9; C: *t*_(31)_ = 0.3, *p* = 0.7].

In summary, the performance comparisons by age suggest that all children showed significantly slower response speed and lower accuracy rates during incongruent and neutral trials than young adults at pre-test. Nine months later at post-test, all children still performed the task more slowly than the young adults, but children who participated in the physical activity intervention did not differ from the adult group in terms of task accuracy. On the other hand, the wait-list control group remained less accurate than the young adults at post-test.

### Functional rois

Table 3 contains mean percent signal change values of each ROI (see Figure 2) at pre-test and post-test in the physical activity intervention and wait-list control groups of children. Table 3 also contains mean percent signal change values in each ROI for the young adults.

**Table 3:** **Mean percent signal change (SD) (range) for the physical activity (PA) and control (C) child groups and the young adult group during incongruent, neutral and NoGo task trials (versus baseline)**.

	**PA pre-test**	**PA post-test**	**C pre-test**	**C post-test**	**Young**
**INCONGRUENT**					
Right anterior prefrontal cortex	0.62 (0.5) (−0.01 to 1.31)^a^	0.19 (0.6) (−1.07 to 1.40)^a^	0.67 (0.5) (−0.13 to 1.71)	0.70 (0.4) (0.00 to 1.26)	0.22 (0.4) (−0.69 to 1.21)
Anterior cingulate cortex	0.56 (0.5) (−0.21 to 1.35)	0.37 (0.4) (−0.53 to 2.12)	0.54 (0.4) (−0.02 to 0.76)	0.38 (0.3) (0.01 to 0.98)	0.38 (0.3) (−0.66 to 1.67)
**NEUTRAL**					
Right anterior prefrontal cortex	0.53 (0.5) (−0.21 to 1.35)	0.45 (0.7) (−0.35 to 1.70)	0.38 (0.3) (−0.02 to 0.76)	0.51 (0.3) (0.01 to 0.98)	0.23 (0.5) (−0.66 to 1.67)
Anterior cingulate cortex	0.75 (0.5) (0.19 to 1.81)	0.52 (0.3) (0.02 to 1.22)	0.44 (0.4) (−0.11 to 0.89)	0.44 (0.3) (−0.28 to 0.89)	0.31 (0.3) (−0.21 to 0.98)
**NOGO**					
Right anterior prefrontal cortex	0.32 (0.6) (−0.35 to 1.70)	0.11 (0.6) (–1.09 to 1.07)	0.38 (0.2) (0.10 to 0.77)	0.51 (0.1) (0.28 to 0.80)	0.26 (0.4) (–0.59 to 0.93)
Anterior cingulate cortex	0.30 (0.5) (−0.45 to 1.67)	0.27 (0.3) (−0.43 to 0.57)	0.18 (0.4) (−0.56 to 0.57)	0.11 (0.3) (−0.35 to 0.46)	0.17 (0.3) (−0.41 to 0.85)

Note: Values that share a common superscript are significantly different at p < 0.05. Effect sizes (Cohen's d) for the right anterior prefrontal cortex: PA pre-test to post-test changes for the right anterior prefrontal cortex: incongruent-ES = 0.78, neutral-ES = 0.13, NoGo-ES = 0.35. C pre-test to post-test changes for the right anterior prefrontal cortex: incongruent-ES = 0.07, neutral-ES = 0.43, NoGo-ES = 0.82.

#### Right anterior prefrontal cortex

**Figure 4:**
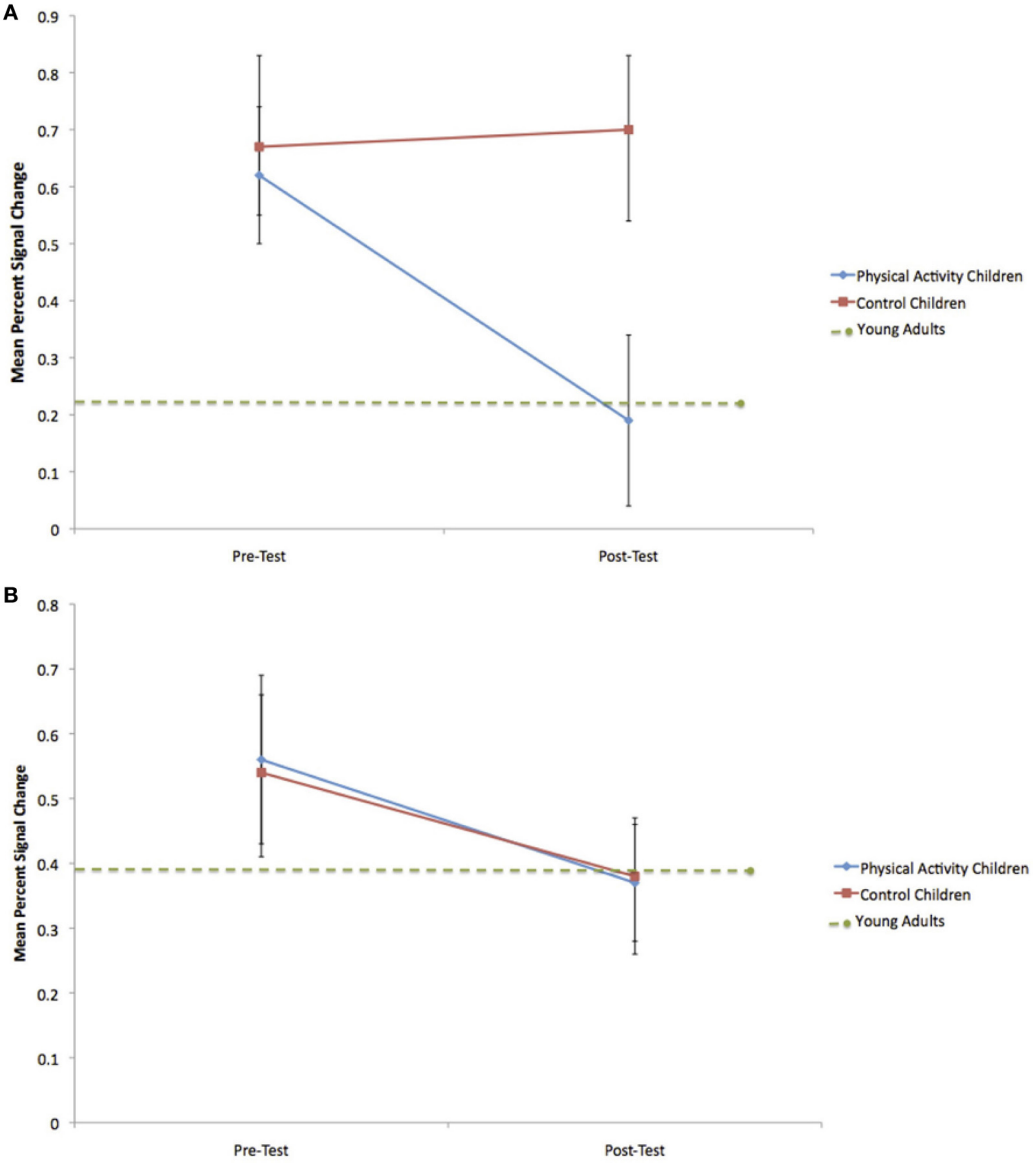
**(A)** Change in mean percent signal change in the right anterior prefrontal cortex during incongruent flanker trials for the physical activity intervention group and wait-list control group. Error bars represent standard error. The decrease in activation for the physical activity intervention group led to mean percent signal change at post-test that did not differ from young adults. **(B)** Change in mean percent signal change in the anterior cingulate cortex during incongruent flanker trials for the physical activity intervention group and wait-list control group. Error bars represent standard error. No significant group differences were observed.

Consistent with predictions, a significant group × time interaction [*F*_(1, 21)_ = 5.4, *p* = 0.03] demonstrated that children in the physical activity intervention group and wait-list control group showed differential changes in fMRI activation in the right anterior prefrontal cortex, from pre-test to post-test. The physical activity intervention group showed a significant decrease in right anterior prefrontal activation across all task conditions from pre-test (*M* = 0.49, *SD* = 0.34) to post-test (*M* = 0.25, *SD* = 0.41). No change in activation from pre-test (*M* = 0.47, *SD* = 0.33) to post-test (*M* = 0.58, *SD* = 0.45) was found for the wait-list control group in the right anterior prefrontal cortex. In an exploratory planned comparison, this change in activation that was found for the physical activity intervention group was driven by activation decreases during the incongruent condition [*t*_(13)_ = 3.5, *p* = 0.004] (see Figure 4A), and no significant within-group changes in neutral or NoGo activation (see Table 3).

Similar to the performance comparisons above, pre-test and post-test brain activation in the right anterior prefrontal cortex of the physical activity intervention group and wait-list control group of children were compared to the activation of the young adults in this ROI. Consistent with predictions, during incongruent trials, which necessitated increased cognitive control, both the physical activity intervention group [*t*_(36)_ = 2.6, *p* = 0.01] and the wait-list control group [*t*_(31)_ = 2.5, *p* = 0.02] had more activation in the right anterior prefrontal cortex compared to the young adults at pre-test (see Table 3). At post-test, activation of the right anterior prefrontal cortex in the physical activity intervention group became statistically equivalent to the young adults [*t*_(36)_ = 0.2, *p* = 0.8] (see Figure 4A), whereas, the wait-list control group still showed activation differences at post-test [*t*_(31)_ = 2.9, *p* = 0.008] (see Table 3). During neutral trials (and NoGo trials), which required less cognitive control than incongruent trials, neither group of children was statistically different in right anterior prefrontal cortex activation at pre-test or post-test (all *p* > 0.05). In sum, children in the physical activity intervention group showed significant decreases in activation in the right anterior prefrontal cortex from pre-test to post-test during incongruent flanker trials, which led to activation patterns that mirrored the patterns of young adults at post-test. Wait-list control children did not show changes in right anterior prefrontal cortex activation from pre-test to post-test and showed significant activation differences from young adults at pre-test and post-test.

It is noteworthy that all children showed adult-like activation during task trials that required less cognitive control (e.g., neutral and NoGo trials). Furthermore, the data suggest that the wait-list control children were unable to upregulate these processes to support task conditions requiring additional control (e.g., incongruent trials). This framework supports previous fMRI and ERP studies in children (Pontifex et al., 2011; Chaddock et al., 2012a), which showed, in cross-sectional studies, that higher fit children and lower fit children had similar brain patterns and performance during trials with low cognitive demands, but only higher fit children were able to maintain performance and adapt neural recruitment to successfully perform on trials with increased cognitive demands.

#### Anterior cingulate cortex

In the ACC, the group × time interaction was not significant [*F*_(1, 21)_ = 0.2, *p* = 0.6] (see Table 3 and Figure 4B). Furthermore, neither the physical activity intervention group nor wait-list control group showed significant differences in activation from the young adult group at pre-test or post-test {all *t* < 1.8, *p* > 0.07, *except* one significant difference for ACC activation during neutral trials between the physical activity group and young adults only at pre-test [*t*_(36)_ = 3.3, *p* = 0.002]}. These findings suggest that brain function in the ACC did not significantly change from pre-test to post-test in children and that children activated the ACC at a similar level to the young adults at both pre-test and post-test.

#### Supplemental results

Two additional regions outside the hypothesized frontal cortex were shown on the *Z* statistic map of average activation during all task conditions for all children, across pre-test and post-test (insula and occipital pole) (see Figure 1). In an 8 mm peak ROI in the insula (voxel coordinates of 59, 71, 40), the group × time interaction was not significant [*F*_(1, 21)_ = 2.9, *p* = 0.13]. Furthermore, neither the physical activity intervention group nor wait-list control group showed significant differences in activation in the insula from the young adult group at pre-test or post-test (all *t* < 2.0, *p* > 0.05). Additionally, in an 8 mm peak ROI in the occipital pole (voxel coordinates of 33, 18, 39), the group × time interaction was not significant [*F*_(1, 21)_ = 0.16, *p* = 0.69]. Furthermore, neither the physical activity intervention group nor wait-list control group showed significant differences in activation in the occipital pole from the young adult group at pre-test or post-test (all *t* < 1.6, *p* > 0.10). These findings suggest that brain function in the insula and occipital cortex did not significantly change from pre-test to post-test in children and that children activated these areas at a similar level to the young adults at both pre-test and post-test.

## Discussion

This study had three main goals. First, this study examined the areas of the brain, especially the prefrontal cortex, associated with a task of cognitive control in children. Research has shown that the frontal cortex is especially involved in cognitive control (Cabeza and Nyberg, 2000; Bunge and Crone, 2009) and development (Gogtay et al., 2004). Second, this study examined whether a 9-month physical activity intervention would influence performance on a task of cognitive control as well as the frontal brain regions involved in processing challenging task demands, relative to a wait-list control group. Third, this study explored whether changes in performance and activation in the physical activity intervention group and wait-list control group from pre-test to post-test mirrored performance and activation in college-aged young adults. Although this study was limited by a relatively small sample size, the results extend investigations of how physical activity and individual differences in aerobic fitness might be associated with improved brain function (via fMRI, ERP) involved in cognitive control in children (Hillman et al., 2005, 2009; Davis et al., 2011; Pontifex et al., 2011; Voss et al., 2011; Chaddock et al., 2012a). The findings provide a foundation for future research to examine, with larger sample sizes, the effect of physical activity on frontal brain function.

Regarding the first goal, two areas of the frontal cortex were found to be associated with the cognitive control task (*Z*-stat > 6), independent of task condition or group assignment. The task-related frontal regions were found in the right anterior prefrontal cortex and the ACC. Both the anterior prefrontal cortex and ACC are known to work together to comprise cognitive control networks (Dosenbach et al., 2007, 2008; Fair et al., 2007). The anterior prefrontal cortex is involved in the maintenance of task context, task goals, and cognitive control over time (i.e., across the trials of a task) (Koechlin et al., 1999; Rushworth et al., 2004; Dosenbach et al., 2006, 2007). For example, Koechlin et al. (1999) demonstrated that the bilateral anterior frontal lobe (i.e., frontal pole) was activated during a task that required participants to keep in mind a main goal while processing and exploring concurrent subgoals. Because such goal maintenance skills are useful for planning and reasoning (Koechlin et al., 1999), it is important to understand how factors such as physical activity may influence brain function of this region during development, a critical period in which the brain matures, learns, and forms connections (Amso and Casey, 2006). The ACC is also known to play a role in cognitive control, via the monitoring of response conflict (often engendered through error production) and signaling the frontal cortex to regulate top-down cognitive control (Botvinick et al., 2001; Dosenbach et al., 2007, 2008). Both of these areas have been found to relate to physical activity and aerobic fitness across the lifespan (Colcombe et al., 2004; Voss et al., 2011; Chaddock et al., 2012a). The present study used a randomized controlled intervention design in children to explore the effects of physical activity on the fMRI brain function of both of these regions.

In regards to the second goal, a significant group × time interaction demonstrated that children in the physical activity intervention group showed significant decreases in fMRI activation in the right anterior prefrontal cortex from pre-test to post-test, whereas the activation patterns in this frontal region in the wait-list control group remained unchanged. It is noteworthy that exploratory planned comparisons revealed that these activation changes in the physical activity intervention group were driven by decreases in activation during incongruent flanker trials that required the greatest challenge to the participants' ability to pay attention and suppress distraction. In fact, relevant to the third goal, the activation decreases in the physical activity intervention group during the incongruent flanker condition led to post-test fMRI patterns in the right anterior prefrontal cortex that did not differ in magnitude from young adult activation. On the other hand, children in the wait-list control group differed from young adults in right anterior prefrontal activation during incongruent flanker trials at both pre-test to post-test.

Together, these group-related and age-related activation patterns raise the possibility that participation in physical activity during childhood can lead to more adult-like recruitment of anterior prefrontal brain areas important for maintenance and goal-oriented cognitive control. Here, improved brain function is associated with decreases in anterior prefrontal cortex activation from pre-test to post-test, which is consistent with the framework that less brain activation reflects more mature brain function, as a number of studies show decreased activation and superior performance on cognitive tasks in adults compared to children (Casey et al., 1997; Durston et al., 2002; Booth et al., 2003; Scherf et al., 2006; Velanova et al., 2008). Behaviorally, exploratory planned comparisons demonstrated that the physical activity intervention group showed within-group performance improvements in terms of both speed and accuracy during incongruent and neutral flanker trials. The incongruent accuracy rates of the physical activity intervention children at post-test also mirrored those of the young adults. In contrast, wait-list control children did not show changes in task performance from pre-test to post-test. These performance differences could be driven by changes in maintenance of task context and task goals with the physical activity intervention, which are functions linked to the anterior prefrontal cortex (Koechlin et al., 1999; Rushworth et al., 2004; Dosenbach et al., 2006, 2007).

In fact, previous studies have demonstrated an association between physical activity, aerobic fitness, and anterior prefrontal brain function involved in goal maintenance across the lifespan (Voss et al., 2010, 2011; Kamijo et al., 2011). This longitudinal intervention study in children extends and strengthens these findings. In children, a cross-sectional fMRI study by Voss et al. (2011) demonstrated that higher fit children showed less activation in a network of brain regions including the anterior prefrontal cortex, coupled with better flanker task performance, relative to lower fit children. An ERP study by Kamijo et al. (2011) also demonstrated that children involved in a physical activity intervention showed larger amplitudes over the frontal scalp regions in the contingent negative variation (CNV), an ERP component known to play a role in cognitive preparation and task maintenance, as well as better working memory performance. In older adults, a physical activity intervention that involved walking 3 days per week, for 1 year, led to changes in functional connectivity in a frontal-executive network (Voss et al., 2010), a network that includes the right and left anterior prefrontal cortex (Dosenbach et al., 2006). The results of the present study contribute to this literature and suggest plasticity of the right anterior prefrontal cortex with prolonged physical activity participation.

No changes in activation for the physical activity intervention group or wait-list control group were found in the ACC. In addition, no differences were observed in the comparison of child and adult ACC activation at pre-test or post-test. Consistent with these findings, a cross-sectional study of the association between aerobic fitness and cognitive control in children did not demonstrate fitness differences in the ACC during incongruent flanker trials (Voss et al., 2011). Further, Chaddock et al. (2012a) also reported few fitness-related activation differences in this area. However, higher fit children (Pontifex et al., 2011), higher fit younger adults (Themanson et al., 2008), and higher fit older adults (Colcombe et al., 2004), as well as older adults involved in a physical activity intervention (Colcombe et al., 2004), have shown smaller ERN amplitudes [an ERP component said to originate in the dorsal portion of the ACC (Dehaene et al., 1994; Carter et al., 1998; van Veen and Carter, 2002; Miltner et al., 2003)], and less ACC activation, respectively, which are associated with performance improvements on a flanker task. Such activation patterns in the ACC are usually interpreted as a reduction in conflict or a lower threshold for the detection and signaling of conflict to the prefrontal cortex, which leads to better error detection. To address this divergent evidence, additional research is needed to better understand different responses to physical activity in children and older adults, how effects in extreme fitness groups (higher fit, lower fit) in cross-sectional studies differ from effects of an intervention with lower fit individuals, as well as how ERP components map onto fMRI activity.

The data also raise the possibility that the two groups of children differed in their cognitive strategies at post-test. Cognitive control strategies are theorized to develop from one that is more rapid and reactive (i.e., reactive control) to one that can flexibly sustain goal-oriented control (i.e., proactive control) (Braver et al., 2007, 2009; Fair et al., 2007). Participation in physical activity during childhood may influence fMRI brain patterns underlying control strategies, specifically the anterior prefrontal cortex (Fair et al., 2007; Paxton et al., 2008). That is, physically active children may learn to maintain a sustained task set during cognitive demands that require selective attention and distraction suppression, which may lead to a more proactive control strategy as well as more accurate and adult-like task performance. This would parallel research that suggests that higher fit children and older adults use a more proactive control neural strategy than lower fit individuals, especially during incongruent flanker task conditions (Colcombe et al., 2004; Pontifex et al., 2011; Voss et al., 2011). Alternatively, children in a wait-list control group may be less able to adapt their task strategy and task set at post-test, and may continue to use a more reactive strategy, given that anterior prefrontal activation and performance on incongruent task trials were unchanged.

These results have important implications for public health and the educational environment. Physical activity opportunities are being reduced or eliminated during the school day as well as decreasing outside the school environment (Troiano et al., 2008). Children are becoming increasingly sedentary and unfit, which leads to an increased risk for disease and obesity (United States Department of Health and Human Services, 2008; Centers for Disease Control and Prevention, 2009), as well as cognitive impairment (Chaddock et al., 2012c). The present study suggests that physical activity is important to the development of the brain and cognition during childhood. These results should raise public awareness of the cognitive benefits of being active and encourage participation in a multicomponent physical activity program such as physical education, classroom activity breaks, and active transport to school (United States Department of Health and Human Services, 2013).

### Conflict of interest statement

The authors declare that the research was conducted in the absence of any commercial or financial relationships that could be construed as a potential conflict of interest.
